# Corrigendum to: DDEL-17. TRIPLE INTRAVENTRICULAR CHEMOTHERAPY FOR TREATMENT OF RELAPSED CHOROID PLEXUS CARCINOMA

**DOI:** 10.1093/neuonc/noad020

**Published:** 2023-02-07

This is a corrigendum to: Grace Lau, Julie Drummond, Nataliya Zhukova, Paul Wood, Lisa Janson, DDEL-17. TRIPLE INTRAVENTRICULAR CHEMOTHERAPY FOR TREATMENT OF RELAPSED CHOROID PLEXUS CARCINOMA, *Neuro-Oncology*, Volume 22, Issue Supplement_3, December 2020, Page iii287, https://doi.org/10.1093/neuonc/noaa222.052

In the originally published online version of this manuscript, Paul Wood was inadvertently omitted from the list of authors.

This error has now been corrected.

